# Verbal fluency as a quick and simple tool to help in deciding when to refer patients with a possible brain tumour

**DOI:** 10.1186/s12883-022-02655-9

**Published:** 2022-04-04

**Authors:** Karolis Zienius, Mio Ozawa, Willie Hamilton, Will Hollingworth, David Weller, Lorna Porteous, Yoav Ben-Shlomo, Robin Grant, Paul M. Brennan

**Affiliations:** 1 Translational Neurosurgery, Centre for Clinical Brain Sciences, University of Edinburgh, Edinburgh, UK; 2 Medical School, Population Health Sciences, University of Bristol, Bristol, UK; 3 College of Medicine and Health, University of Exeter, Exeter, UK; 4 Usher Institute of Population Health Sciences and Informatics, University of Edinburgh, Edinburgh, UK; 5 GP Lead for Cancer and Palliative Care, NHS Lothian, Edinburgh, UK; 6 Department of Clinical Neurosciences, NHS Lothian, Western General Hospital, Edinburgh, UK; 7 Centre for Clinical Brain Sciences, University of Edinburgh, Little France Crescent, Edinburgh, EH16 UK

**Keywords:** Brain tumour, Neurocognitive test, Early diagnosis of cancer, Verbal fluency

## Abstract

**Background:**

Patients with brain tumours often present with non-specific symptoms. Correctly identifying who to prioritise for urgent brain imaging is challenging. Brain tumours are amongst the commonest cancers diagnosed as an emergency presentation. A verbal fluency task (VFT) is a rapid triage test affected by disorders of executive function, language and processing speed. We tested whether a VFT could support identification of patients with a brain tumour.

**Methods:**

This proof-of-concept study examined whether a VFT can help differentiate patients with a brain tumour from those with similar symptoms (i.e. headache) without a brain tumour. Two patient populations were recruited, (a) patients with known brain tumour, and (b) patients with headache referred for Direct-Access Computed-Tomography (DACT) from primary care with a suspicion of a brain tumour. Semantic and phonemic verbal fluency data were collected prospectively.

**Results:**

180 brain tumour patients and 90 DACT patients were recruited. Semantic verbal fluency score was significantly worse for patients with a brain tumour than those without (*P * <  0.001), whether comparing patients with headache, or patients without headache. Phonemic fluency showed a similar but weaker difference. Raw and incidence-weighted positive and negative predictive values were calculated.

**Conclusion:**

We have demonstrated the potential role of adding semantic VFT score performance into clinical decision making to support triage of patients for urgent brain imaging. A relatively small improvement in the true positive rate in patients referred for DACT has the potential to increase the timeliness and efficiency of diagnosis and improve patient outcomes.

**Supplementary Information:**

The online version contains supplementary material available at 10.1186/s12883-022-02655-9.

## Introduction

Timely diagnosis of a brain tumour is challenging. Over a third of patients consult their primary care doctor on 3 or more occasions before diagnosis [1]. Brain tumours are amongst the commonest cancer type to be diagnosed as an emergency presentation in the UK [2], and many of these patients will have seen their primary care doctor previously.

The first symptoms experienced by patients with a brain tumour are often non-specific: such symptoms are more frequently associated with a benign non-tumour condition [3]. For example, headache is the commonest symptom of brain tumours in adults [4], and is present in over half of patients at the time of diagnosis. Headache also occurs in 4.4% of all primary care consultations [5], so is not specific to brain tumours. Other symptoms, such as subtle memory/cognitive or behavioural changes, may accompany headaches associated with a brain tumour, but are rarely noticed by patients [6]. Unsurprisingly, there is often a delay in patient presentation to primary care, referral for investigation and ultimately in diagnosis. In a recent analysis of brain tumour patients from the National Audit of Cancer Diagnosis in Primary Care (NACDPC) we found that the longest pathway intervals (time from onset of symptoms to referral to a specialist for investigation) were for headache only (median 61 days), memory symptoms (median 62 days), and behavioural/cognitive symptoms (median 39 days) [7]. This compares to an average pathway interval from symptom onset to actual diagnosis for all tumours examined in the English National Cancer Registry of 45 days [8]. Non-specific symptoms are most likely to be associated with diagnostic delay.

It is difficult to determine which patients with non-specific symptoms are most at risk of having a brain tumour and thus should be prioritised for urgent imaging. Symptom-based referral guidelines for suspected brain tumour have been developed to support decision making in primary care. Amongst patients referred for brain imaging based on these guidelines, a brain tumour is identified at most approximately 3% of the time [9, 10]. This strategy results in many false positive subjects, where symptom-based decision making predicts the possibility of a brain tumour, but there is no tumour on brain imaging. Since approximately two-thirds of patients with a brain tumour are diagnosed in the Emergency Department when their symptoms deteriorate, many having already previously seen their GP. The symptom-based referral approach misses many cases.

A new strategy is needed to assist health care professionals in determining which patients with these non-specific symptoms are most at risk of having a brain tumour and should be prioritised for urgent brain imaging.

A verbal fluency task (VFT) [11] is a rapid triage test that is affected by disorders that affect executive function, language and processing speed. The VFT is often a component of comprehensive neuro-cognitive batteries for patients with brain tumours [12–14], but has not been investigated in isolation in relation to brain tumour diagnosis. Unlike a formal neuro-cognitive assessment that is time consuming and requires specialist training and knowledge to administer and interpret, the VFT could be quickly and easily administered in primary care. In a study of cognition and capacity, using the Addenbrooke’s Cognitive Examination-revised (ACE-R), VFT was demonstrated to predict of lack of capacity to give consent to surgery by brain tumour patients [15]. We hypothesized the VFT might therefore also be used to help differentiate patients with brain tumours from those who do not have a brain tumour where both patient groups have similar symptoms. This “proof of concept” study examined our hypothesis. A conference abstract of this work was previously published.

## Methods

### Participants

We included two patient populations, (a) patients with a known brain tumour who presented with both headache and non-headache symptoms, and (b) patients with headache who had been referred from primary care to a Direct Access Computer Tomography (DACT) service with a suspicion of a brain tumour. Eligible patients were fluent in English. The study was performed at the regional clinical neurosciences centre, Western General Hospital, Edinburgh. An appropriate institutional review board approved the recruitment of these patient populations. The data collected on the tumour cases was approved by the South-East Scotland Research Ethics Service (17/SS/0019). The control data were collected as part of an NHS Lothian approved quality improvement project that was auditing the DACT pathway. The study utilised routinely collected clinical data and both the Scotland Research Ethics Service and NHS Lothian determined than no formal patient consent was required, because the study analysed data that is collected as part of routine clinical care. Study methods complied with the ethical principles set out in the Declaration of Helsinki.

The tumour patients were all aged over 16 years attending assessment for surgical treatment of a newly diagnosed brain tumour, based on radiographic appearances on gadolinium enhanced magnetic resonance imaging (MRI), between 19 August 2016 and 14 February 2018. All patients with a new radiological diagnosis of a brain tumour in South East Scotland are discussed at our regional multidisciplinary meeting. Only the most profoundly unwell patients are not offered either biopsy or debulking surgery (approximately 20%) [16], so this sample is representative of the referred population with a brain tumour. Patients with a severe language impairment that affected communication and was considered therefore to affect verbal fluency test performance were excluded from this study at a screening phase. This was because they could not understand or complete assessments. Final tumour diagnosis was confirmed by histopathology.

The comparator group were patients aged over 16 years with headache, with or without other symptoms (e.g. cognitive, behavioural or personality change), referred from primary care for direct access computed tomography (DACT) between 3 May 2017 and 9 February 2018 whose final diagnosis excluded a brain tumour. It was a convenience sampling strategy with a 1:2 ratio of non-tumour to tumour group. The DACT pathway has been available to primary care doctors in Lothian since 1999 and gives access to brain imaging to exclude a significant intracranial pathology where the primary care doctor judges that waiting for a specialist referral will take too long, but immediate hospital admission via the emergency department is not necessary. Since 2016 the referral proforma has included a requirement to assess verbal fluency.

### Procedure

All testing as part of routine care was administered by a clinical research fellow (KZ). This was conducted prior to completion of a DACT in the control group. For patients with a brain tumour, assessment was performed at the pre-operative outpatient visit. The test was conducted in the English language and participants had a good command of English. Semantic (category) [17] verbal fluency was assessed by asking each participant to name as many different animals as they could within 60 s. All responses were recorded verbatim in the order in which they were generated. Repetitions were discounted. Phonemic verbal (letter) [18] fluency was then assessed by asking patients to generate as many different words beginning with a letter “P”, excluding proper names (city, country, person) and repetitions of the same word with different endings (e.g. put, putting). Three scores were calculated for each fluency task: 1) the total number of correct words generated, 2) the mean cluster size (see appendix 1) of each group of animals e.g. domestic animals or zoo animals, and 3) the raw number of switches between clusters, as originally suggested by Troyer [19, 20]. Mean cluster and switching are qualitative aspects of fluency tests that are thought to specify contribution of semantic and executive processes during task production, respectively.

#### Other covariates

We recorded age and gender and used the Scottish Index of Multiple Deprivation score (SIMD) as an ecological marker of socioeconomic status. This is an official measure for deprivation using routine census data developed by Scottish Government and based on current postcode of residence. These were grouped into quintiles to derive an ordinal variable with 1 being the most deprived to 5 being the least deprived. Tumour characteristics including lobe, laterality and size were derived from radiological reports accessible via electronic patient records (EPR). Histological diagnosis was based on formal pathology report.

### Statistical analysis and sub-groups

Baseline characteristics of tumour and non-tumour patient groups were compared using t-test and chi-square tests, as appropriate. Group differences on each verbal fluency measure were analysed using the ANCOVA test, with age group (16–59, 60–79, ≥ 80 years) and gender as covariates. Normality was assessed by visual inspection of histograms of characteristics of interest. Pearson correlation coefficients were used to look at the linear association between total score and qualitative fluency measures.

We decided that the most appropriate clinical comparison was to compare tumour patients with a history of headache against DACT patients (all of whom had headache) as our primary analysis. Headache is the most common symptom associated with a brain tumour, but is much more likely to be associated with a non-tumour diagnosis. We also undertook several sensitivity analyses to examine whether any findings were robust and also to enhance precision where the sample size was increased. We therefore undertook additional sensitivity comparisons with tumour patients (a) with headache as their first symptom (relevant to a patient’s early presentation to primary care, when a tumour diagnosis is often overlooked) and (b) all tumour patients (relevant to scaling application of this test across presentation with non-specific symptoms) versus the DACT patient group.

In our analysis we decided to look at good performance, i.e. a high verbal fluency score, as a positive test which we assumed would help discriminate controls from brain tumour cases. In this scenario, a false positive is someone with a good VFT score who does turn out to have a brain tumour after further investigation. Clinically, a positive test combined with the prior probability of a brain tumour could be used to avoid unnecessary immediate referral for neuroimaging. We used a receiver operating characteristic (ROC) curve and the area under the curve (AUC) to determine the optimal balance of sensitivity versus specificity when discriminating tumour patients from non-tumour patients, using different cut-points of performance on the VF tasks. The raw positive predictive value (PPV) and negative predictive value (NPV), positive likelihood ratio and negative likelihood ratio were than manually calculated. We then repeated the PPV and NPV calculation adjusting for disease incidence in the population. We decided a priori to do this with either only tumour patients with headache or the whole sample of brain tumour cases depending on the sensitivity analyses finding little difference between the case groups. In addition, a multivariable logistic regression model, with adjustment for the covariates age group, gender and deprivation score, with a fluency cut-off value as a dummy variable and group (brain tumour or DACT) as a binary outcome variable, was used to calculate the diagnostic odds ratio, and corresponding 95% confidence intervals (CI). All analyses were performed with SPSS version 21. We also examined whether verbal fluency performance was influenced by tumour characteristics such as brain tumour grade, site and size.

## Results

Nine patients were excluded at the screening stage; seven tumour patients—two patients were unable to converse in English, two had impairment of language function, three were too unwell to participate. Two DACT patient were unable to participate, because of hearing impairment.

180 tumour patients and 90 DACT patients were recruited. Clinical characteristics of the two patient groups, and of the tumours, are detailed in Table 1. Ninety-one (50.5%) of the tumour patients were from Lothian. All DACT patients were from Lothian.

**Table 1:** Patient and Controls baseline characteristics

	Brain tumour patients *N* = 180 (%)	Controls *N* = 90 (%)	*p* value
Age in years, M (SD)	56.8 (13.5)	53.6 (19.5)	0.16
16–59	93 (52)	53 (59)	0.028
60–79	84 (46)	31 (34)	
≥ 80	3 (2)	6 (7)	
Gender (N, %)			
Male	86 (48)	31 (34)	0.037
Female	94 (52)	59 (66)	
Social deprivation index (N, %)			
1	28 (15.7)	11 (12.2)	0.8
2	43 (24.2)	26 (28.9)	
3	32 (18.0)	15 (16.7)	
4	35 (19.7)	15 (16.7)	
5	40 (22.5)	23 (25.6)	
Karnofsky Performance Status median (range)	90 (50–100)	N.A	N.A
Tumour histology, N (%)		N.A	N.A
High grade glioma (WHO III-IV)	77 (42.8)		
Cerebral metastasis	28 (15.6)		
Meningioma	42 (23.3)		
Low grade glioma (WHO I-II)	14 (7.8)		
CNS Lymphoma	5 (2.8)		
Other	14 (7.8)		
Tumour lobe, N (%)		N.A	N.A
Frontal	68 (37.8)		
Temporal	34 (18.9)		
Parietal	18 (10)		
Occipital	13 (7.2)		
Cerebellar	16 (8.9)		
Multiple	15 (5.4)		
Other	16 (8.9)		
Tumour laterality, N (%)		N.A	N.A
Left	80 (44.4)		
Right	64 (35.6)		
Bilateral	22 (12.2)		
Midline	14 (7.8)		
Maximum size T1 (axial), mean (mm) (SD)	41.1 (15.1)	N.A	N.A
Midline shift, N (%)		N.A	N.A
< 5 mm	119 (66.1)		
5–10 mm	31 (17.2)		
> 10 mm	21 (11.7)		
Unknown	9 (5)		

Patients with high grade glioma (HGG) (e.g. glioblastoma (GBM)) comprised the highest proportion (42.8%), followed by meningioma (23.3%). The brain tumour patients were slightly older but this may have been due to chance. There were a higher proportion of women in the DACT group than the tumour group (66% versus 52% respectively, *p* = 0.037). There were no major differences in the proportion of patients in each social deprivation quintile (*p* = 0.8). Most tumours were in the frontal lobe (37.8%) and were left sided (44.4%). Tumour size, based on axial measurement on T1 weighted imaging, ranged from 6 to 80 mm, (mean 41.1 mm). Patients with HGG had the largest tumours on average at diagnosis (44.3 mm).

### Verbal fluency in patients with a history of headache

Assessment of normality was performed by Visual inspection of the distribution of verbal fluency scores across two groups (patients and controls) analysed with histograms and QQ plots which allowed us to assume normal distribution.

102 patients with tumour and 90 DACT patients were included in this first analysis, comparing tumour patients with a history of headache against DACT patients (all of whom had headache). Tumour patients had reduced total score and cluster score with semantic (*p* < 0.001) and phonemic (*p* = 0.001, *p* = 0.018) verbal fluency (Table 2). The standardised difference demonstrated a relatively large difference for semantic total score between cohorts (-0.97) and a medium effect size for phonemic total score (-0.47). By contrast, the results for switching were very similar between the groups.

**Table 2:** Adjusted mean scores* on semantic and phonemic verbal fluency measures for cases and controls: a) cases with a history of headache, b) headache as the first symptom

	**Patients**	**Controls**	**Mean diff (95% CI)**	**p value**	**Effect Size (Cohen’s d)**
**Verbal fluency, mean (SD)**					
**Cases with a history of headache**	***N*** **= 102**	***N*** **= 90**			
**Animals**					
Total	13.0 (5.04)	17.8 (5.05)	-4.9 (-6.32,-3.42)	** < 0.001**	-0.97
Mean cluster	1.1 (0.98)	1.7 (0.95)	-0.6 (-0.9,-0.3)	** < 0.001**	-0.62
Switch	5.9 (3.30)	6.9 (3.31)	-0.9 (-1.9,0.4)	0.06	-0.30
**Letter P**					
Total	8.8 (4.75)	11 (4.74)	-2.2 (-3.6,-0.86)	**0.001**	-0.47
Mean cluster	0.29 (0.33)	0.4 (0.38)	-0.1 (-0.2,-0.02)	**0.018**	-0.32
Switch	6.5 (3.99)	7.7 (4.00)	-1.15 (-2.3,-0.0)	**0.05**	-0.31
**Cases with headache as 1**^**st**^** symptom**	***N*** **= 56**	***N*** **= 90**			
**Animals**					
Total	13.6 (5.07)	17.9 (5.03)	-4.2 (-5.9,-2.5)	** < 0.001**	-0.85
Mean cluster	1.1 (1.07)	1.7 (0.95)	-0.6 (-0.9,-0.2)	**0.001**	-0.62
Switch	6.3 (3.33)	6.9 (3.31)	-0.6 (0.6)	0.34	-0.18
**Letter P**					
Total	9.3 (4.71)	10.9 (4.71)	-1.7 (-3.3,-0.1)	**0.037**	-0.34
Mean cluster	0.3 (0.37)	0.4 (0.36)	-0.1 (-0.2,0.5)	0.24	-0.27
Switch	6.7 (4.03)	7.7 (4.00)	-1.0 (-2.4,0.4)	0.16	-0.25

When tumour patients were restricted to those with headache as their first symptom (56 cases) compared to the DACT patients who also had headache as their primary symptom (Table 2, Supplementary Table 1), the results for total and mean cluster score with semantic fluency were similar (*p* < 0.001, *p* = 0.001). The total score for phonemic fluency was of weak significance (*p* = 0.037), but the mean cluster size for phonemic fluency was consistent with chance (*p* = 0.24).

### *Adjusted for age group and gender

The same pattern of results was found when we used all the cases (180) regardless of whether they had experienced a headache or not (Table 3). The significance values varied.

**Table 3:** Adjusted mean scores on semantic and phonemic verbal fluency measures for all tumour patients and DACT patients

	**Patients (N = 180)**	**Controls (N = 90)**	**Mean diff (95% CI)**	**p value**	**Effect Size (Cohen’s d)**
**Verbal fluency, mean (SE)**					
**Animals**					
Total	12.6 (5.21)	17.7 (5.21)	-5.1 (-6.32,—3.77)	** < 0.001**	-0.98
Mean cluster	1.1 (0.94)	1.7 (0.85)	-0.55 (-0.79, -0.32)	** < 0.001**	-0.62
Switch	5.6 (3.22)	6.9 (3.22)	-1.29 (-2.1,—0.46)	**0.002**	-0.4
**Letter P**					
Total	8.45 (4.61)	10.99 (4.62)	-2.53 (-3.7, -1.35)	** < 0.001**	-0.55
Mean cluster	0.29 (0.41)	0.4 (0.38)	-0.11 (-0.21, -0.01)	**0.025**	-0.28
Switch	6.38 (3.94)	7.68 (3.95)	-1.29 (-2.3, -0.29)	**0.012**	-0.33

### Verbal fluency to discriminate between tumour and non-tumour patients

We sought to identify whether a threshold fluency task score might discriminate brain tumour patients from patients without brain tumours. Semantic verbal fluency total score was analysed because this was more discriminating than phonemic verbal fluency.

**Fig. 1:**
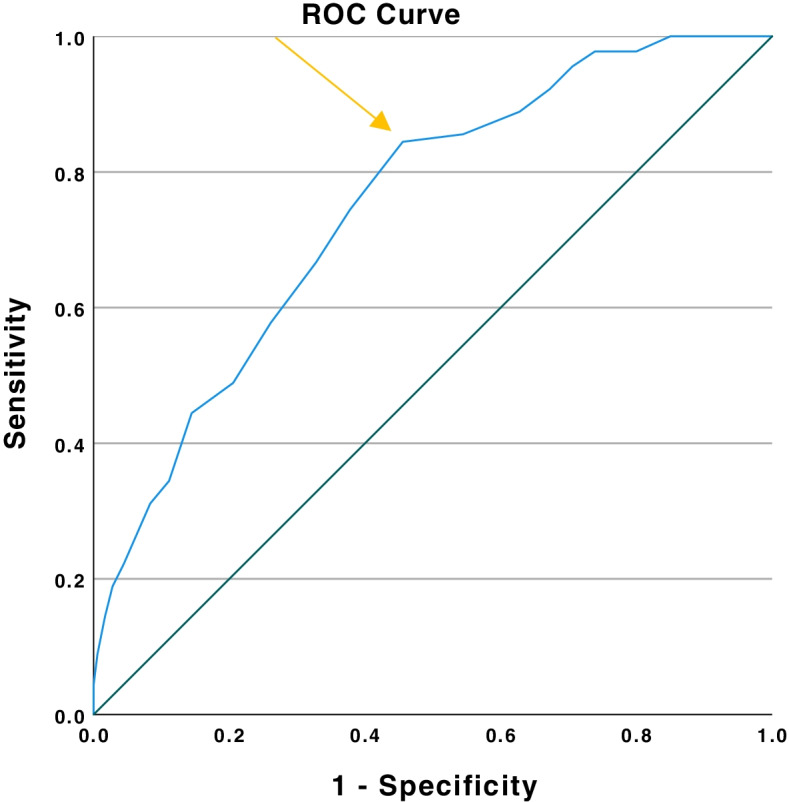
Receiver Operating Curve of sematic verbal fluency scores from total tumour cohort. The arrow indicates the score of 14

Given that brain tumour patients do not just present with headache, we used the receiver operating characteristic (ROC) curve for the cohort of all 180 tumour patients, as the results for headache only and all cases were very similar, compared to the DACT patients. The area under the curve (AUC) was 0.75, (*p* < 0.001) indicating the value of using verbal fluency as a diagnostic test (0.5 no discrimination, 0.7 to ≤ 0.8 acceptable, > 0.8 to ≤ 0.9 excellent, > 0.9 outstanding) [21]. We chose a threshold score of 14 or more animals in 1 min as this looked like the inflection point in the ROC though other values could be used depending on the relative importance of false positives versus false negatives (Fig. 1, Supplementary Table 2). This threshold had the following diagnostic utilities for differentiating non tumour from brain tumour subjects: 84.4% sensitivity (correctly identifying non-tumour patients because of good performance), 54.5% specificity (patients with a tumour who performed badly), 48.1% positive predictive value (PPV) (percentage of patients with good VFT who did not have a tumour), 87.5% negative predictive value (NPV) (percentage of patients with poor VFT who did have a tumour), positive likelihood ratio 1.85 (increased likelihood of not have a brain tumour if VFT is good) and negative likelihood ratio 0.29 (decreased likelihood of not have a brain tumour if VFT is poor). A positive likelihood ratio of between 1 to 2 is regarded as a modest improvement whilst a negative likelihood ratio of between 0.2 to 0.3 would be regarded as good with an approximate 25 to 30% change in probability [22]. The PPV and NPV reported above are misleading as they reflect the sampling strategy that was used in this study (i.e. 1:2 ratio of non-tumour to tumour) rather than what would be expected in primary care. A previous UK general practice database found that amongst patients aged over 50 years, the incidence of a malignant brain tumour amongst new headache presentations was 28 per 10,000 patients [9] or a prior probability of 0.28% (the converse is that the probability that the patient with a headache doesn’t have a brain tumour is 99.7%). Applying these risks to the marginal totals and using the same sensitivity and specificity as above, we would now get a revised PPV for a positive test of 99.8% and a NPV of 0.97%. This shows that a positive test slightly increases the likelihood that this is not a brain tumour but a negative test is of more concern. Despite the low absolute risk of having a tumour, a poor performance on the fluency task has more than tripled the probability in relative terms.

Multivariable logistic regression, adjusting for age group, gender and deprivation quintile score, found that patients scoring at or above the cut-point of ≥ 14 had a nearly 8 times higher odds of not having a brain tumour (adjusted OR 7.84, 95%CI 3.89 15.8, *p* < 0.001) (Table 4). This association was even stronger when we repeated the analysis restricted to only the 102 tumour patients with a history of headache (adjusted OR 9.33, 95% CI 4.2, 20.9, *p* < 0.001).

**Table 4:** Logistic regression for association between age group, gender or area deprivation score and semantic verbal fluency in the cohort of 180 tumour patients. 95% confidence intervals indicated. Odds ratio of not having a brain tumour

Variable	Odds Ratio	95% confidence Interval
**Semantic verbal fluency value**		
0–13	**Reference**	
≥ 14	7.84	3.9–15.8
**Age**		
16–59	**Reference**	
60–79	0.9	0.5–1.66
80 +	6.68	1.3–33.5
**Gender**		
Male	**Reference**	
Female	1.87	1.04–3.35
**Deprivation score**		
1	**Reference**	
2	1.2	0.5–3.1
3	1.3	0.5–3.7
4	0.85	0.3–2.4
5	1.16	0.44–3.1

Correlation analysis (Table 5) was performed to examine the relationship between the total animal and letter P scores and the qualitative fluency parameters, namely mean clustering and switching. Analyses were undertaken separately for each patient and control group.

**Table 5:** Pearson’s r correlation coeficients between Animals and Letter *P* – Total scores and qualitative fluency parameters in tumour patients and controls

	**Animals—Total**		**Letter** ***P*—Total**	
	Patients	Controls	Patients	Controls
**Mean cluster size**	0.34*	0.18^ m^	0.13	-0.1
**Switches**	0.78*	0.42*	0.92*	0.88*

^*^p < 0.001

^m^*p* < 0.1

For tumour patients, both clustering and switching highly correlated with a total score on an animal fluency task, while for a letter P task only switching was significantly associated with a total score. For controls, only switching was significantly associated with a total score for both animal and phonemic fluency, although for animal test, a trend for clustering was observed. Thus, at this stage, correlation patterns for the control group is considered to indicate a trend and remains to be confirmed with a larger dataset.

### Verbal fluency associations with brain tumour grade, site and size

The reduction in verbal fluency task performance was most marked in patients with high grade gliomas (HGG) (mean total 10.8), cerebral lymphoma (mean total 10.9) and cerebral metastases (mean total 12.3), the three most malignant brain tumours. Patients with HGG had significantly lower total semantic scores compared to meningioma (mean total 14.3) (mean difference -3.44, *p* = 0.018), and ‘other’ intracranial tumours (mean total 16.8) (mean difference -5.95, *p* = 0.002).

A frontal lobe tumour location, in contrast to a non-frontal location was associated with significantly lower fluency performance total scores for both semantic (mean = 11.6 vs 13.3, *p* = 0.04) and phonemic (mean = 7.3 vs mean = 9.2, *p* = 0.007) tasks. Tumours in the left hemisphere had a larger effect on phonemic than semantic total fluency scores (left vs right: mean difference -2.3, *p* = 0.004 and -1.9, *p* = 0.04, respectively). Both left and right hemisphere lesions impacted significantly on semantic verbal fluency. Increasing tumour size correlated with lower semantic total (*r* = -0.22, *p* = 0.003) and cluster (*r* = -0.22, *p* = 0.003) score. There was little size correlation for any measure on phonemic fluency.

## Discussion

This study is the first to compare performance of a verbal fluency task between a large brain tumour patient population and a comparator population of patients with headache referred for direct access brain imaging from primary care to exclude significant intracranial pathology. We have demonstrated that verbal fluency tasks, both semantic (animal) and phonemic (letter “P”), differed between the two patient populations. However, the effect size for semantic total score was nearly twice that of letter P total score suggesting that one should only use the former. In patients where the primary care doctor has a low suspicion of a significant intracranial pathology, and the patient has a high VF score, there is more reassurance that a period of further clinical observation may be justified. However, a poor performance, in the absence of another cause, is of more concern to the primary care doctor and may lower their threshold for urgent imaging.

Our data demonstrate that the semantic verbal fluency test is more discriminatory than phonemic fluency. We found no added value for the use of switching and cluster size than using the simple total count. Semantic fluency task is generally considered easier to perform and people are able to generate more responses in a minute, because of its ecological validity, or daily familiarity [23]. Animal fluency is also less influenced by level of education[24]. A tendency for worse performance on semantic compared to phonemic fluency is also reported in Alzheimer’s disease^25.^. This lack of specificity between brain tumours and dementia in less problematic for the patient, as clinicians often order brain imaging in patients with suspected dementia though it would be less urgent.

Cognitive symptoms in patients with a brain tumour may be subtle and noticed by patients [26], though relatives may perceive these changes several months before diagnosis [27]. Self-awareness of cognition can be influenced by cognitive problems affecting an ability to retain and elaborate such information [27]. A lack of insight into the nature or urgency of symptoms may prevent a patient from seeking help early [27]. Semantic verbal fluency as a cognitive test may therefore be particularly useful in patients presenting with headache and reports of memory or cognitive changes. This is consistent with our previous finding that patients presenting with symptoms of headache, or behavioural and memory changes, have the longest time from symptom onset to brain tumour diagnosis [7].

The majority of patients presenting to primary care with non-specific symptoms such as headache without neurological signs will not have a significant intracranial pathology. Only 1–3% of current brain images performed through a DACT route identify a brain tumour and many of these are incidental findings [28]. Taken along with a good clinical history, the semantic verbal fluency task may therefore be helpful in the primary care consultation to quickly test cognition, for example in a patient presenting with a concerning new onset headache. A combination of worrying symptoms and poor verbal fluency score could support decision making, as to whether to request urgent brain imaging. Many patients with a low verbal fluency will still not have a brain tumour, but our expectation is that the addition of a poor verbal fluency test would be an improvement over the current situation. As important, in patients with headache, where there is a low index of suspicion for tumour, a good performance on verbal fluency task may provide some reassurance and support a decision for continued observation.

We regard this a proof of concept study rather than a definitive diagnostic test accuracy study and there were several limitations. The tumour patients were assessed after their diagnosis had been made, and pre-operatively, so their performance may not be the same as what it would have been had they been tested when they first presented in primary care. The DACT patients in our study were referred for brain imaging to exclude a significant intracranial pathology or to provide reassurance. The DACT route is biased towards patients with less worrying symptoms than those who would have been sent directly to the emergency department. The verbal fluency test will not be discriminatory for brain tumour in patients with a pre-existing dementia diagnosis, as this also affects VF test performance.

We compared the test performance in tumour patients without headache against all tumour patients and our observations appear to be generalizable. Our ‘control’ population had headache symptoms and were of enough concern to their primary care doctor to warrant imaging. This contrasts with most previous studies that have compared neurocognitive performance in brain tumour patients with healthy controls [29, 30]. Our study design better reflects the patient population seen within primary care where headache is a frequent and non-specific symptom and therefore reduces the likelihood of “spectrum bias”.

All the DACT patients lived in Lothian, whilst some of the tumour patients came from outside the Lothian area, but we don’t believe that these geographical differences would have biased the performance on the fluency task. The importance of education on verbal fluency performance is still debated, with some studies [31] showing significant effect, whilst others failing to demonstrate it [19, 31]. We were not able to adjust for educational level, however, our ecological analysis of area deprivation did not suggest any major differences by this proxy measure of socioeconomic status. Individuals with higher educational level may perform better than normal despite having some decline due to a brain tumour thus masking an effect. This is the reason why the Montreal Cognitive Assessment (MoCA) test used to screen for dementia adjusts its score for educational level. It is therefore possible that an adjusted VFT score might be a better differentiator than the crude score. Future studies should empirically test this hypothesis, as well as examine other patient factors that may affect test performance, such as a previous history of alcohol and drug abuse.

## Conclusion

Diagnosis of a brain tumour is challenging and the decision as to whether a patient is at risk of a brain tumour requires careful clinical assessment. We have demonstrated the potential role of adding semantic verbal fluency task score performance into clinical decision making around referral for urgent brain imaging. Further research in this area will be required and the optimal cut-off point for the verbal fluency score will depend on a more thorough analysis of the costs and benefits of balancing sensitivity and specificity. A prospective diagnostic test accuracy study in a patient group at risk for a brain tumour diagnosis would provide stronger evidence as to the utility of the verbal fluency test over and above clinical signs, symptoms and the prior probability. The impact of the test should be evaluated in terms of time to diagnosis, and how this translates into clinical care and patient outcomes before we can adopt this test into clinical guidelines.

## Supplementary Information

**Additional file 1:** **Supplementary Table 1. **ANCOVA Model tables for each outcome verbal fluency outcome measure. **Supplementary Table 2. **Sensitivity and Specificity of cut off points on receiver operating curve forsemantic verbal fluency.

## Data Availability

The datasets analysed during the current study are available from the corresponding author on reasonable request.
