# Supplementary material for: Verbal fluency as a quick and simple tool to help in deciding when to refer patients with a possible brain tumour
Source: BMC Neurol. 2022 Apr 4;22:127. doi: 10.1186/s12883-022-02655-9 (PMC8978365; doi:10.1186/s12883-022-02655-9)
Supplement: Supplementary file 1 — Additional file 1: Supplementary Table 1. ANCOVA Model tables for each outcome verbal fluency outcome measure. Supplementary Table 2. Sensitivity and Specificity of cut off points on receiver operating curve forsemantic verbal fluency. [file 12883_2022_2655_MOESM1_ESM.docx]

Supplementary Table 1. ANCOVA Model tables for each outcome verbal fluency outcome measure

ANCOVA model: cases with a history of headache for **Animal Total**

|  | df | F | sig | Partial Eta squared |
| --- | --- | --- | --- | --- |
| Group | 1 | 43.7 | <0.001 | 0.19 |
| Age | 1 | 12.9 | <0.001 | 0.06 |
| Gender | 1 | 0.65 | 0.4 | 0.003 |
| Error | 188 |  |  |  |

Acova model: cases with a history of headache for **Letter P**

|  | df | F | sig | Partial Eta squared |
| --- | --- | --- | --- | --- |
| Group | 1 | 10.4 | 0.001 | 0.053 |
| Age | 1 | 1.3 | 0.26 | 0.007 |
| Gender | 1 | 0.9 | 0.34 | 0.005 |
| Error | 188 |  |  |  |

Ancova model: cases with headache as 1^st^ symptom for **Animal Total**

|  | df | F | sig | Partial Eta squared |
| --- | --- | --- | --- | --- |
| Group | 1 | 23.6 | <0.001 | 0.14 |
| Age | 1 | 6.8 | <0.01 | 0.05 |
| Gender | 1 | 0.23 | 0.63 |  |
| Error | 142 |  |  |  |

ANCOVA model: cases with headache as 1^st^ symptom for **Letter P**

|  | df | F | sig | Partial Eta squared |
| --- | --- | --- | --- | --- |
| Group | 1 | 4.4 | 0.04 | 0.03 |
| Age | 1 | 0.01 | 0.9 | 0 |
| Gender | 1 | 1.5 | 0.2 | 0.1 |
| Error | 142 |  |  |  |

ANCOVA model: for all tumour cases for **Animal Total**

|  | df | F | sig | Partial Eta squared |
| --- | --- | --- | --- | --- |
| Group | 1 | 57.1 | <0.001 | 0.18 |
| Age | 1 | 18.1 | <0.001 | 0.1 |
| Gender | 1 | 1.1 | 0.3 | 0 |
| Error | 266 |  |  |  |

ANCOVA model: for all tumour cases for **Letter P**

|  | df | F | sig | Partial Eta squared |
| --- | --- | --- | --- | --- |
| Group | 1 | 17.9 | <0.001 | 0.06 |
| Age | 1 | 2.3 | 0.13 | 0.01 |
| Gender | 1 | 2.9 | 0.1 | 0.01 |
| Error | 266 |  |  |  |

Supplementary Table 2. Sensitivity and Specificity of cut off points on receiver operating curve for semantic verbal fluency.

| Positive if greater than or equal to* | Sensitivity | Specificity (%) |
| --- | --- | --- |
| .5000 | 1.000 | 2.8 |
| 1.5000 | 1.000 | 4.4 |
| 2.5000 | 1.000 | 5.0 |
| 3.5000 | 1.000 | 6.1 |
| 4.5000 | 1.000 | 7.8 |
| 5.5000 | 1.000 | 10.6 |
| 6.5000 | 1.000 | 15.0 |
| 7.5000 | .978 | 20.0 |
| 8.5000 | .978 | 26.1 |
| 9.5000 | .956 | 29.4 |
| 10.5000 | .922 | 32.8 |
| 11.5000 | .889 | 37.2 |
| 12.5000 | .856 | 45.6 |
| 13.5000 | .844 | 54.4 |
| 14.5000 | .744 | 62.2 |
| 15.5000 | .667 | 67.2 |
| 16.5000 | .578 | 73.9 |
| 17.5000 | .489 | 79.4 |
| 18.5000 | .444 | 85.6 |
| 19.5000 | .344 | 88.9 |
| 20.5000 | .311 | 91.7 |
| 21.5000 | .222 | 95.6 |
| 22.5000 | .189 | 97.2 |
| 23.5000 | .144 | 98.3 |
| 24.5000 | .089 | 99.4 |
| 25.5000 | .044 | 1.0 |
| 26.5000 | .011 | 1.0 |
| 28.0000 | .000 | 1.0 |
